# Robust Hydrophobic Surfaces from Suspension HVOF Thermal Sprayed Rare-Earth Oxide Ceramics Coatings

**DOI:** 10.1038/s41598-018-25375-y

**Published:** 2018-05-03

**Authors:** M. Bai, H. Kazi, X. Zhang, J. Liu, T. Hussain

**Affiliations:** 1 Faculty of Engineering, University of Nottingham, Nottingham, NG7 2RD UK; 2 School of Engineering and Materials Science, Queen Mary University of London, London, E1 4NS UK

## Abstract

This study has presented an efficient coating method, namely suspension high velocity oxy-fuel (SHVOF) thermal spraying, to produce large super-hydrophobic ceramic surfaces with a unique micro- and nano-scale hierarchical structures to mimic natural super-hydrophobic surfaces. CeO_2_ was selected as coatings material, one of a group of rare-earth oxide (REO) ceramics that have recently been found to exhibit intrinsic hydrophobicity, even after exposure to high temperatures and abrasive wear. Robust hydrophobic REO ceramic surfaces were obtained from the deposition of thin CeO_2_ coatings (3–5 μm) using an aqueous suspension with a solid concentration of 30 wt.% sub-micron CeO_2_ particles (50–200 nm) on a selection of metallic substrates. It was found that the coatings’ hydrophobicity, microstructure, surface morphology, and deposition efficiency were all determined by the metallic substrates underneath. More importantly, it was demonstrated that the near super-hydrophobicity of SHVOF sprayed CeO_2_ coatings was achieved not only by the intrinsic hydrophobicity of REO but also their unique hierarchically structure. In addition, the coatings’ surface hydrophobicity was sensitive to the O/Ce ratio, which could explain the ‘delayed’ hydrophobicity of REO coatings.

## Introduction

Rare-earth oxide (REO) ceramics have received increasing attention during the last few years owing to their intrinsic hydrophobicity and robustness to harsh environment^1,2^. The intrinsic hydrophobicity of REOs arises from the unique electronic structure of the RE metals atoms where the unfilled 4*f* orbitals are shielded from interactions by the filled 5*s*^2^*p*^6^ outer shell^1^. This extraordinary feature makes REOs stand out from the common metals and ceramics which are generally hydrophilic with contact angle (CA) less than 90°. More importantly, unlike polymeric modifiers, e.g. polytetrafluoroethylene (PTFE, or Teflon®), which are used as conventional hydrophobic coatings, REOs exhibited sustained hydrophobicity after high temperature exposure to up to 1000 °C for 2 h and abrasive-wear test^1^. Accordingly, these robust hydrophobic surfaces obtained from REOs ceramics are seen to be incredibly useful for a wide range of applications including anti-icing^3–6^, anti-corrosion^7–9^, drag reduction^10–12^, and dropwise condensation^13–16^, etc. So far, many known techniques have been applied to deposit thin-films of REOs for hydrophobic applications, including atomic layer deposition (ALD)^17^, sputtering^18,19^, and cathodic electrodeposition^20,21^, etc. REO ceramics have also been used to fabricate super-hydrophobic surfaces (CA > 150°) by incorporating micro and nanoscale hierarchical surface textures by depositing a thin layer of REOs onto a textured surface (e.g. nanograss-covered cubical microposts)^1^ or directly texturing the hydrophobic REOs by laser machining^22^. This approach faces the challenges of machining brittle ceramics and fragile silicon substrates.

In 2016, Cai *et al*.^23^ first manufactured super-hydrophobic REO coatings with hierarchically structured topography using precursor solutions as feedstock in a plasma spray deposition process, which is also known as solution precursor plasma spray (SPPS). After vacuum treatment at 1 Pa for 48 hours, the as-sprayed coatings showed a CA value up to 65% higher than on bulk REO surfaces. A recently developed technology, namely suspension high velocity oxy-fuel thermal spraying (SHVOF) was first introduced for the preparation of super-hydrophobic hierarchically-textured TiO_2_/hexagonal boron nitride (h-BN) composite coatings on stainless steel by Zhang *et al*.^24^. Although TiO_2_ is widely known as intrinsically hydrophilic, the resultant TiO_2_/h-BN coatings exhibited super-hydrophobic behaviour when the addition of h-BN reaches 10 wt. % due to the presence of hierarchical nano-texture on the surface. SHVOF also offers a rapid and economical method to produce large hydrophobic surface areas on a variety of substrates. SHVOF uses suspensions as feedstocks instead of solution precursor, which enables the use of submicron and nano-powders to form unique nano-structured coatings with higher deposition efficiency^25–27^. In addition, during spraying, particle injection is directly inside the combustion chamber and thus a considerably good heat transfer of the particles can be realised, which enables complete melting and homogenisation of feedstock powders. Lastly, SHVOF torches can reach very high gas velocities (>2000 m/s)^28^, and the substrates are typically cooled with compressed air jets, which all contribute to rapid cooling of the molten droplets. This results in a significantly high nucleation rate as well as a negligibly low growth rate of crystallites as inhibited by the short residence time due to rapid acceleration in the flame and rapid cooling upon impact, all of which contribute to the formation of nano-crystallized coatings with significant improvement in density, strength and durability^29–31^.

**Figure 1:**
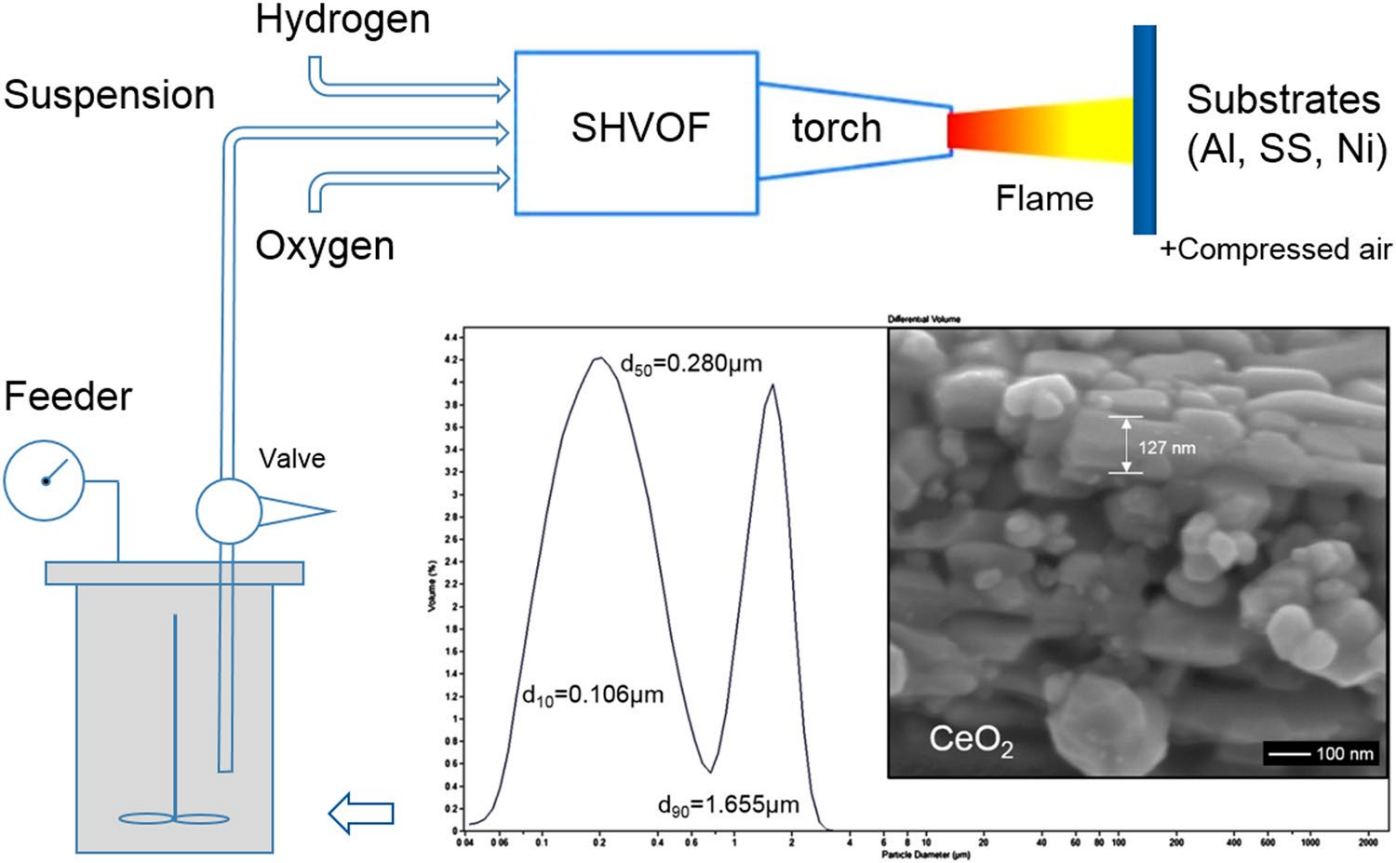
An overview of the suspension HVOF process for spraying CeO_2_ coatings on metallic substrates from suspension feedstock containing CeO_2_ sub-micron particles.

In this study, SHVOF was used to deposit REO coatings with intrinsic hydrophobicity and hierarchically structured surface topography on a selection of commonly used metallic substrates, i.e. aluminium, stainless steel and nickel-based alloys, which can be found in various applications but differ significantly in hardness. CeO_2_ (ceria) has been chosen for study as it is one of the most extensively studied REO materials and also an important product for catalytic application^32^. A schematic drawing of the SHVOF system is shown in Fig. 1 and the CeO_2_ particles dried from the suspension feedstock exhibit granular shapes. It has a particle size range of around 50–200 nm with a d_50_ of 0.280 μm in the suspension feedstock. Detailed characterizations of the as-sprayed coatings were undertaken with main focuses on coatings’ surface topography, microstructure phase, crystallite size and surface hydrophobicity using field emission gun scanning electron microscopy (FEG-SEM), surface profilometry, x-ray diffraction (XRD) with Rietveld Refinement and contact angle (CA) measurement. We aim not only to demonstrate the potential of SHVOF technique for industrial preparation of robust water-repellent REO coatings on various metallic components; but also find out the mechanism for the improvement in surface hydrophobicity of SHVOF sprayed REO coatings.

## Results

### Surface hydrophobicity

**Figure 2:**
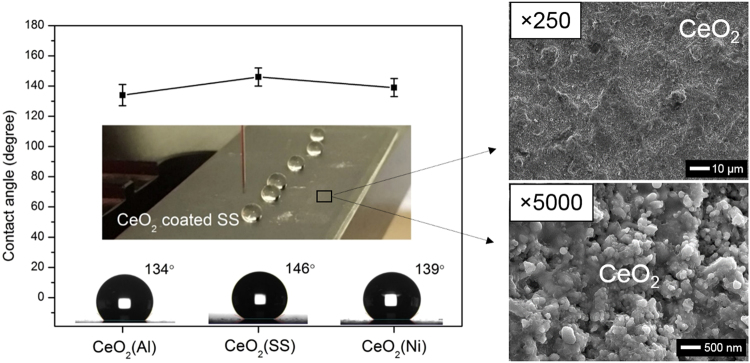
CA measurements on the CeO_2_ coatings on Al, SS and Ni substrates and SEM images showing the typical surface morphology of the CeO_2_ coatings under high magnifications.

Figure 2 shows the contact angle (CA) measurement of the as-sprayed CeO_2_ coatings on the three substrates with an inset photo showing the non-wetting water droplets on the CeO_2_ coated SS. All three coatings exhibited superior surface hydrophobicity with average CA values over 130–140°. Although the static CA measurement with conventional sessile-drop method coupled with the tangent line entails unavoidable errors in determining the air-solid base line due to the smoothness problem and substrate tilting^33^, it has been clearly seen that the CA results in this study are comparable to those in the literatures^23,24^ that reported super-hydrophobicity with CA values of over 150°. More importantly, the CA values on the SHVOF sprayed CeO_2_ coatings are 30–40% higher than those measured on the sintered CeO_2_ pellets with smoother surface^1^. The significant increase of CA values was believed to be attributed to the hierarchically structured surface topography as obtained from the plasma/thermal spraying^23,24^. SEM images on the right show typical surface morphology of the CeO_2_ coatings under high magnifications. A lamellae structure with fully deformed molten droplets is observed on the CeO_2_ coatings showing well-merged and sintered particles with significantly reduced surface area. The formation of these nano-sized or micro-sized features from thermal spray is mainly caused by differing particle trajectories in the flame and by various droplet sizes being formed from the disintegration of primary droplets of suspension^24^. Moreover, the coatings’ surface morphology is found to be dependent on the substrates even though all coatings are sprayed under the same condition, which leads to a variation in surface hydrophobicity.

### Surface morphology

**Figure 3:**
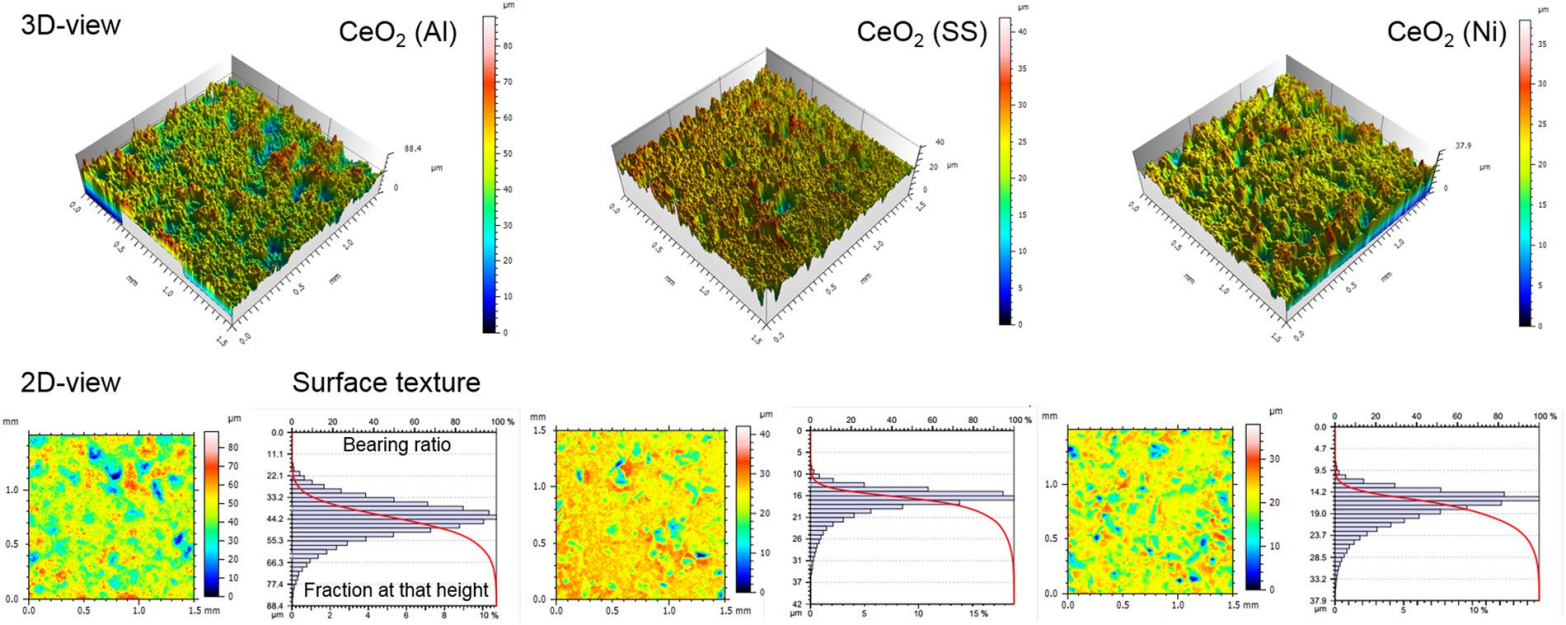
Quantitative characterization of the surface morphology of the CeO_2_ coatings on Al, SS and Ni substrates with 3D views, 2D views and amplitude density function.

Figure 3 shows the quantitative characterization of the surface morphology of the as-sprayed CeO_2_ coatings on the three substrates including the 3D views, 2D views and Abbott-Firestone curves. The unique surface morphology of the SHVOF coatings can be described by the surface roughness (i.e. Sa, Sq) and surface texture (i.e. Ssk, Sku) according to the distribution of surface heights and the spatial distribution of peaks and valleys across the surface as listed in Table 1. With the highest CA values, the CeO_2_ (SS) coatings have the lowest Sq and Sa values indicating the smoothest surface, in combination with the strongest surface texture owing to the highest Sku indicating a ‘spiky’ surface and the lowest Ssk indicating the least symmetrical distribution of heights. While the other two coatings have rougher surfaces and weaker surface texture, which could be described as relating to a ‘bumpy’ surface. It is worth noting that the surface slopes in 2D and 3D views appear much steeper than they really are due to the different magnifications used to represent the dataset in the horizontal and vertical directions. The formation of the distinctive surface morphology is affected by the mechanical properties of the substrates upon the impact of the molten droplets with high kinetic energy during spraying. More detailed characterization results of the as-sprayed CeO_2_ coatings on the three substrates including the FEG-SEM images showing the surface and cross-sectional microstructure, XRD analysis of the phases and crystallize sizes, and discussions on the spraying mechanism can be found in the Supplement Information (See Figures S1–S3).

**Table 1:** Quantitative characterization of the coatings surface and contact angle measurements.

	CeO_2_ (Al)	CeO_2_ (SS)	CeO_2_ (Ni)
Sq (µm, Root-mean-square height)	9.56	3.60	3.81
Sa (µm, Arithmetic mean height)	7.33	2.55	2.91
Ssk (Skewness)	−0.511	−1.62	−1.13
Sku (Kurtosis)	3.86	7.34	4.76
CA (degree, Contact angle)	134	146	139

### Surface effect

**Figure 4:**
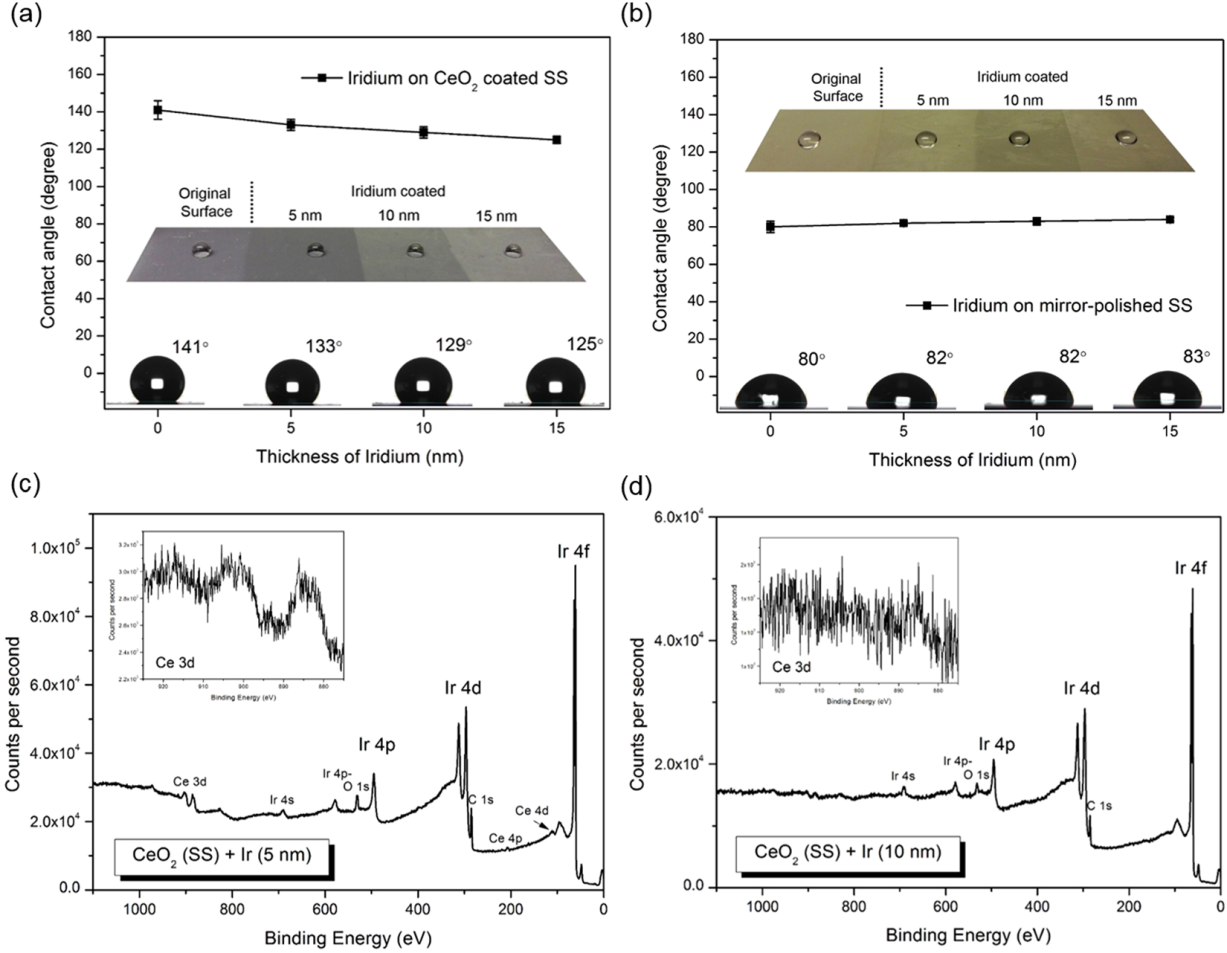
CA measurements on Iridium coated surface of the (**a**) CeO_2_ coated SS; and (**b**) mirror polished SS to examine the effect of the surface morphology of REO coatings on their hydrophobicity, in combination with XPS analysis on the Iridium coated surface with a thickness of (**c**) 5 nm and (**d**) 10 nm to confirm that Iridium has fully covered the CeO_2_ underneath.

A series of experiments were also conducted to examine the effect of the unique surface of SHVOF thermal sprayed CeO_2_ coatings on their surface hydrophobicity as shown in Fig. 4. A precisely controlled Iridium nano-sputter was used to apply a thin Iridium coating on the same coating surface with a highly sensitive film thickness monitor. Iridium provides fine grains (sub-nanometer) films that are more favourable for FEG-SEM high resolution imaging analysis than the others. The aim was not only to rule out the effect of REO material on the surface hydrophobicity by applying a different material with higher surface energy (hydrophilic metal), but also retain the delicate surface morphology of SHVOF coatings. Figure 4(c,d) shows the XPS spectra of the Iridium coated surface with inset figures of the high-resolution XPS spectrum of Ce 3d indicating the full coverage of Iridium over CeO_2_ coatings. With the deposition of over 5 nm of Iridium, the existence of Ce is nearly undetectable. In Fig. 4(a), the CA values drops to around 130° when the thickness of Iridium is 5 nm but no significant change was observed with thicker Iridium coatings. The effect of the REO underneath should be ruled out by the replacement of Iridium, and the CA value of 125–130° could then be considered as the sole contribution of the hierarchical microstructure on the surface. For comparison, Iridium coatings were applied on the mirror-polished SS as shown in Fig. 4(b), and the CA measurements show constant values at around 80°. It demonstrates that the unique hierarchical structure of SHVOF coatings has significantly improved the surface hydrophobicity by contributing to an over 50% increase of CA. These results confirmed that the near-super-hydrophobicity of the SHVOF REO coatings was contributed by both the intrinsic hydrophobic property of REO and the unique hierarchical structure from thermal spraying.

### Surface chemistry

**Figure 5:**
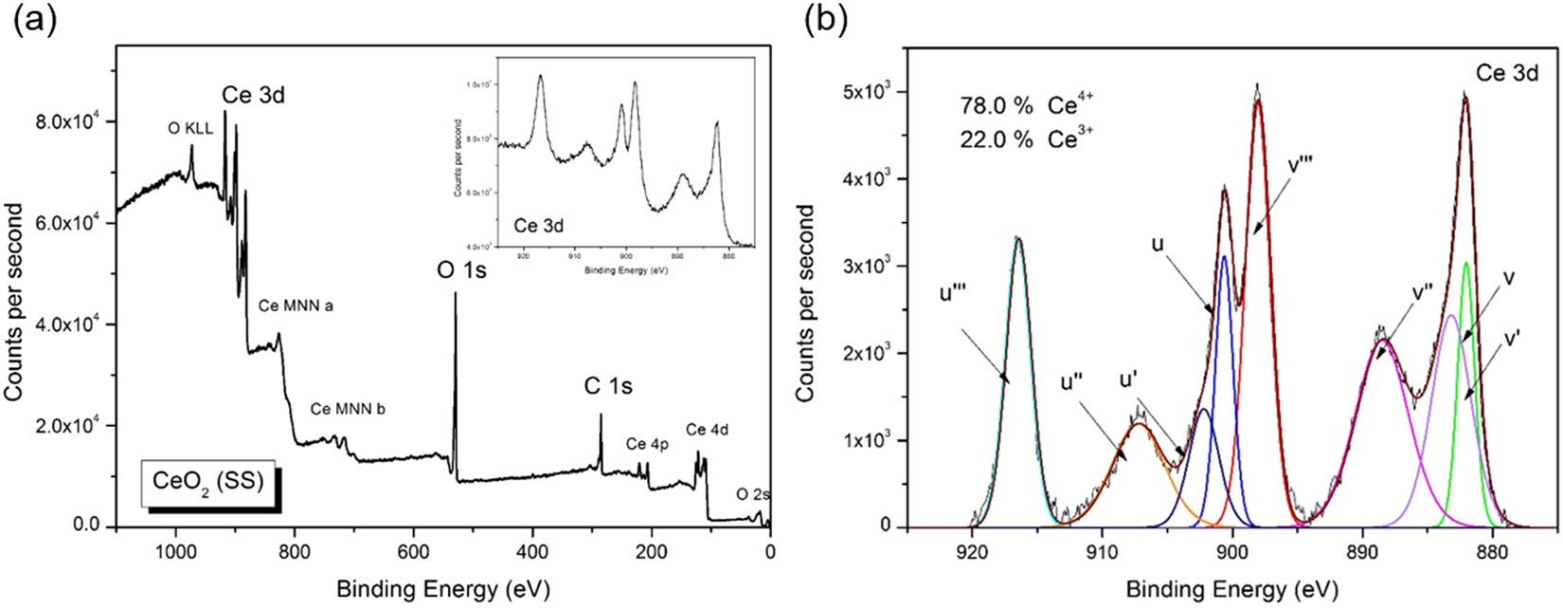
(**a**) XPS survey scan on the as-sprayed CeO_2_ coatings; and (**b**) High-resolution XPS spectrum of Ce 3d peaks with detailed peak deconvolutions and quantification results.

Despite the unique hierarchically structured surface that would contribute to the improvement in surface hydrophobicity of REO coatings, there is another important issue that should not be over looked, i.e. the “delayed” hydrophobicity of the plasma/thermal sprayed REO coatings. This phenomenon was first reported by Cai *et al*.^23^ on the SPPS sprayed REOs coatings, that the as-sprayed coating surface was initially hydrophilic (CA ≈ 0°), but after vacuum treatment at 1 Pa for 48 hours the CA value increased dramatically to over 150°. We have observed similar phenomenon that the coatings immediately after spray were hydrophilic with low CA values (<90°) but then increased to over 130° after 4 weeks left in the ambient air. This transition is believed to be largely dependent on the surface oxygen-to-metal (e.g. O/Ce) ratio, which was proved by Khan *et al*.^19,34^ through rigorous investigations on the wettability of sputtered CeO_2_ surface as a dependency of O/Ce ratios using X-ray Photoelectron Spectroscopy (XPS) in a ultra-high vacuum (UHV) environment. Khan^19^ reported that an O/Ce ratio higher than 2.0 can negatively affect the hydrophobicity as it provides more sites for hydrogen bonding with water. Figure 5 shows the full XPS spectrum and high-resolution spectrum of Ce 3d with detailed deconvolutions into constituent satellite peaks and identified using the nomenclature proposed by Burroughs *et al*.^35^. The XPS peaks were associated with the Ce^4+^ or the Ce^3+^ oxidation state. Peaks v, v″, v″′, u, u″ and u″′ were associated with the Ce^4+^ state, while v^0^, v′, u^0^ and u′ were associated with the Ce^3+^ state. The relative concentrations of Ce^4+^ and Ce^3+^ were determined using the following equations^36^.

1 $${\boldsymbol{A}}({\boldsymbol{C}}{{\boldsymbol{e}}}^{3+})={\boldsymbol{A}}({{\boldsymbol{v}}}_{0})+{\boldsymbol{A}}({\boldsymbol{v}}^{\prime} )+{\boldsymbol{A}}({{\boldsymbol{u}}}_{0})+{\boldsymbol{A}}({\boldsymbol{u}}^{\prime} )$$

2 $${\boldsymbol{A}}({\boldsymbol{C}}{{\boldsymbol{e}}}^{4+})={\boldsymbol{A}}({\boldsymbol{v}})+{\boldsymbol{A}}({\boldsymbol{v}}^{\prime\prime} )+{\boldsymbol{A}}({\boldsymbol{v}}\prime\prime\prime )+{\boldsymbol{A}}({\boldsymbol{u}})+{\boldsymbol{A}}({\boldsymbol{u}}^{\prime\prime} )+{\boldsymbol{A}}({\boldsymbol{u}}\prime\prime\prime )$$

3 $$\begin{array}{cc} \% \,{\boldsymbol{C}}{{\boldsymbol{e}}}^{{\boldsymbol{y}}+}=\frac{{\boldsymbol{A}}({\boldsymbol{C}}{{\boldsymbol{e}}}^{{\boldsymbol{y}}+})}{{\boldsymbol{A}}({\boldsymbol{C}}{{\boldsymbol{e}}}^{3+})+{\boldsymbol{A}}({\boldsymbol{C}}{{\boldsymbol{e}}}^{4+})}\times 100 & ({\boldsymbol{y}}={\bf{3}},{\bf{4}})\end{array}$$

*A*(*x*) above refers to the peak area (and corresponding peak intensity) as determined from the XPS spectra. As calculated, there is 78% Ce^4+^ and 22% Ce^3+^, corresponding to a lattice O/Ce ratio of 1.89 based on the conservation of valence state. This is lower than the stoichiometric value indicating a deficient oxygen and less tendency to form hydrogen bonds with interfacial water and hence a better surface hydrophobicity. The “delayed” hydrophobicity is believed to be caused by the partial reduction in state from Ce^4+^ to Ce^3+^ with time, which is consistent with previous observations^19,37,38^. It was observed by Khan^19^ that the Ce^4+^ contribution decreased from 89.8% to 72.8% after overnight relaxation in UHV which directly led to an increase of CA values from 15° to 104°. Therefore, in view of all these observations, it can be concluded that the change in cerium’s chemical state caused an overall decrease in the surface O/Ce ratio, which ultimately affected the surface hydrophobicity of REO coatings.

To conclude, robust hydrophobic surfaces on a selection of metallic substrates (namely Al, SS and Ni) were obtained from the deposition of thin CeO_2_ coatings (3–5 μm) by SHVOF thermal spraying using an aqueous suspension with a solid concentration of 30 wt.% sub-micron CeO_2_ particles (50–200 nm). Several important conclusions can be drawn as follows,

1. Near super-hydrophobicity was observed with 30–40% increase of CA than that of smooth surface. The use of thin Iridium deposition (5–15 nm) on the surface clearly demonstrated that the improvement was achieved mainly by their unique surfaces with hierarchical structure.
2. The substrates determined the coatings’ hydrophobicity, microstructure, surface morphology, and deposition efficiency, etc. The coatings on SS performed the best among the three with the highest contact angle, smoothest surface and strongest surface texture.
3. The coatings’ surface hydrophobicity was also sensitive to the O/Ce ratio and the ‘delayed’ hydrophobicity of CeO_2_ coatings was caused by the partial reduction in state from Ce^4+^ to Ce^3+^ with time, which ultimately led to a slow transition from hydrophilic to hydrophobic.

## Materials and Methods

### Suspension and sample preparation

A water-based CeO_2_ suspension (MilliDyne, Finland) with a solid concentration of 30 wt.% was used in this study. Particle size distribution was measured using laser diffractometer (LS13320, Beckman-Coulter, USA). Suspension was mixed for 90 mins using a mechanical stirrer at a speed of 940 rpm to achieve a homogeneous suspension. The suspension was sprayed onto 3 different types of substrates with a dimension of 60 × 25 × 2 mm^3^ including (1) Aluminium alloy (1050A), (2) stainless steel (AISI 304) and (3) Nickel Alloy (NIMONIC C263), which are referred to as Al, SS, and Ni, respectively (See Table 2). Substrates were grit blasted with F100 brown alumina particles (0.125–0.149 mm) under 3 bar pressure, and cleaned by an ultrasonic acetone bath to remove any embedded alumina particles.

**Table 2:** Composition and properties of the metallic substrates^*^.

Type	Al	SS	Ni
Alloy	1050A Aluminium	AISI 304 stainless steel	Nimonic C263
Composition (wt.%)	Al-0.4Fe-0.25Si	Fe-19.0Cr-9.3Ni-0.05C	Ni-20Co-20Cr-6Mo
Density (g/cm^3^)	2.79	7.9	8.36
Melting point (°C)	640–650	1400–1455	1300–1355
Hardness (Vickers)	22	129	292
Tensile strength (MPa)	105–145	520–700	630–940
Thermal expansion (10^−6^/K)	24	17.2	10.3
Modulus of elasticity (GPa)	71	193	221
Thermal conductivity (W/m·K)	222	16.2	11.7

^*^Data is provided by the supplier. Mechanical properties were measured at room temperatures.

### SHVOF Thermal spraying

A modified UTP/Miller Thermal HVOF system with a direct injection at the centre of the gas-mixing block was used to spray the suspension. The suspension injector had a diameter of 0.3 mm to inject the suspension into the centre of the combustion chamber. A 22 mm long combustion chamber with 100 mm long barrel nozzle was used in this study. The suspension was fed using a pressurised 2 L vessel equipped with a mechanical stirrer to ensure uniform dispersion of the nano-particles in solution and consistent flow onto the substrate without clogging the nozzle. The pressure of the feeding system was fixed at 5 bar during the spray. The gun was mounted on a z-axis traverse unit in front of the rotating carousel and it was set to a stand-off distance from the surface of the substrate of 85 mm. The substrates were mounted onto a carousel rotating at 73 rpm with a vertical axis of rotation. The substrates were cooled using compressed air jets during and after the spray. The flame heat power was 110 kW with a hydrogen fuel flow rate of 1650 scfh (equals to 775 L/min) and oxygen flow rate of 715 scfh (equals to 336 L/min). Hydrogen was used as the combustion fuel to achieve a cleaner flame and to reduce any hydrocarbon combustion products in the resulting coating, as any contamination might affect the hydrophobic behaviour of coatings. The theoretical flame heat power for each spray was calculated using standard combustion formulae.

### Characterizations methods

SEM with JEOL in-lens Schottky field emissions source (FEG-SEM, JEOL 7100F, USA) was used to examine the morphology of the feedstock powder dried from the suspension, as well as the surface morphology and cross-sectional microstructure of the as-sprayed coatings. The coating thickness was analysed by Image Pro-Plus (6.0, Media Cybernetics, Rockville, USA) software. Hydrophobicity of the surfaces was characterized using a contact angle goniometer (FTA200, First Ten Angstroms, Inc., Portsmouth, VA, USA) with pumping out rate of 1 µL/s. Quantitative characterization of the coatings surface was carried out using an Alicona InfiniteFocus G5 focus variation microscopy (FVM) with 20x objective lens (vertical resolution 50 nm). Data was further analysed using MountainsMap® Premium software version 7 (Digital Surf, Besançon, France) to generate 3D views, 2D views, and detailed surface roughness and texture results. Surface roughness were measured by stylus surface profilometry (Talysurf CLI 1000 Profilometer, Leicester, U.K.) with a resolution of 40 nm. Quorum 150T S turbomolecular-pumped coating system (Quorum Technologies, UK) was used to sputter-coat samples with iridium, and the coating thickness was precisely controlled by a Film Thickness Monitor at a deposition rate of 3 nm/min. XRD analysis was conducted using a Siemens D500 (Germany) with Cu*Kα* produced at 40 kV and 25 mA. The 2*θ* diffraction angle range was from 10° to 90° with step width of 0.05° and time per step of 2 seconds. Quantitative Rietveld refinement (TOPAS V5 software package) was employed to determine the crystallite size based on the principles of whole powder pattern modelling (WPPM)^39^. The penetration depth of the X-ray through CeO_2_ was calculated according to the literature^40^. The surface chemistry of the coating samples was analysed using a Kratos AXIS Supra X-ray photoelectron spectroscopy (XPS, Kratos Analytical Limited, UK) equipped with a GCIS (Kratos minibeam 6), and Al Kα X-ray source (1486.6 eV). The settings for the GCIS were the same for all experiments, 10 kV Ar^+^_1000_ with rastered areas of 1 mm^2^. The photoelectron acquisition was performed on each sample at three positions with a wide survey scan and high-resolution scans: O 1s, C 1s, Ce 3d and Ir 4f. The data analysis was carried out by the CasaXPS software (Version 2.3.18PR1.0) with Kratos sensitivity factors (RSFs) to determine relative atomic concentrations from the peak areas. The energy scale of the spectra was corrected for sample charging.

## Electronic supplementary material

Supplementary Information
