# Supplementary material for: Robust Hydrophobic Surfaces from Suspension HVOF Thermal Sprayed Rare-Earth Oxide Ceramics Coatings
Source: Sci Rep. 2018 May 3;8:6973. doi: 10.1038/s41598-018-25375-y (PMC5934356; doi:10.1038/s41598-018-25375-y)
Supplement: Supplementary file 1 — Supplementary Information [file 41598_2018_25375_MOESM1_ESM.doc]

**Supplementary information**

Robust Hydrophobic Surfaces from Suspension HVOF Thermal Sprayed Rare-Earth Oxide Ceramics Coatings

M. Bai^1^, H. Kazi^1^, X. Zhang^2^, J. Liu^1^, T. Hussain^1^*

^1^ Faculty of Engineering, University of Nottingham, Nottingham, NG7 2RD, UK

^2^ School of Engineering and Materials Science, Queen Mary University of London, London E1 4NS, UK

*Corresponding author: tanvir.hussain@nottingham.ac.uk; +441159513795

*Surface and cross-sectional microstructure*


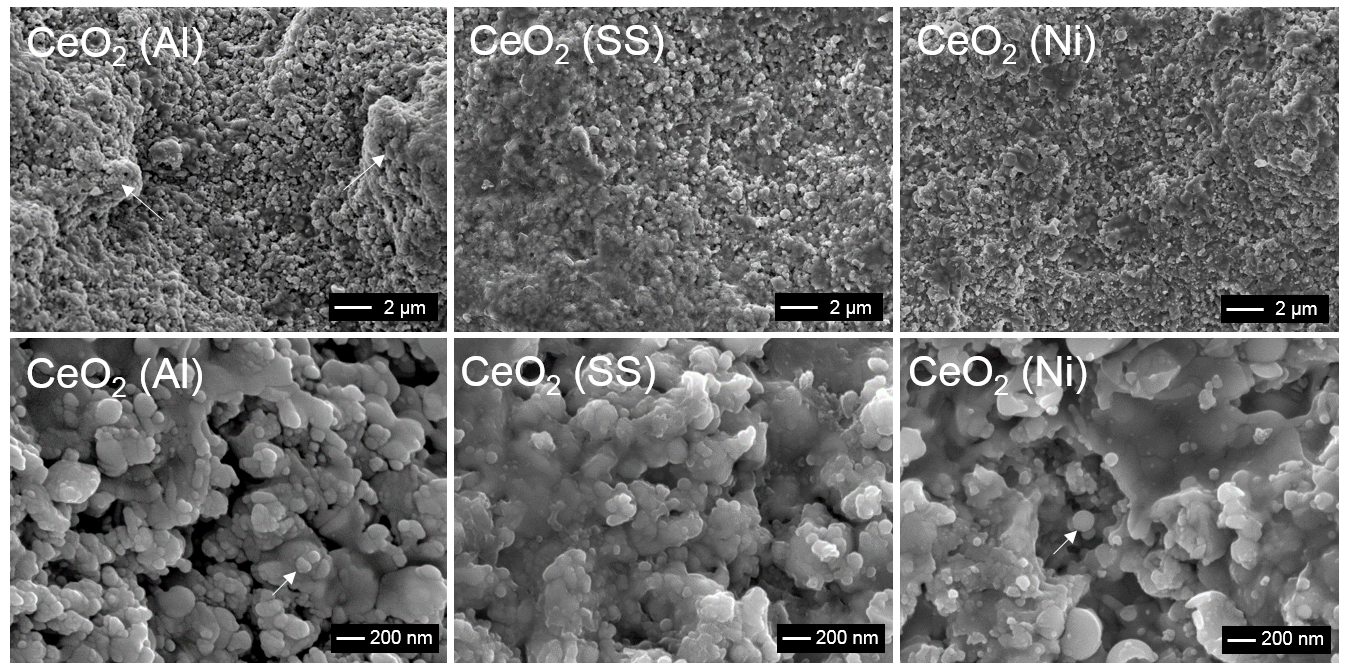


Figure S1. FEG-SEM images showing the surface morphology of the as-sprayed CeO_2_ coatings on Al, SS and Ni substrates under low (top row) and high (bottom row) magnifications.

Figure S1 shows the surface morphology of the as-sprayed CeO_2_ coatings on Al, SS and Ni substrates under low and high magnifications in FEG-SEM with randomly selected areas and representative surface features. First of all, at low magnifications, the CeO_2_ (Al) surface shows distinctive “hump-like” features that are scattered throughout its surface (marked by white arrows in the figure); while the other two have smoother and more uniform surfaces, which agrees with the surface roughness measurements. The “hump-like” or “protuberance-like” features have also been reported on SHVOF sprayed TiO_2_ coatings^1^ and Al_2_O_3_ Coatings^2^ with several possible explanations. Nevertheless, the formation of humps on the CeO_2_ (Al) is most likely caused by the severe undulation of the Al substrates due to the low yield strength of Al at high temperatures. More detailed surface features can be revealed from the high magnification images acquired from the central area, which exhibited distinctive shapes of sprayed particles that have all gone through deformation and solidification upon impact on different substrates. While individual non-deformed particles are observed on the CeO_2_ coated surfaces of both Al and Ni (marked by white arrows in the figures), indicating insufficient kinetic energy of the molten droplets upon impact on the substrates^3,4^. This is possibly attributed to the significant differences in hardness of these three substrates as shown earlier in Table 2. To be more precise, Al substrates might be too soft, which absorbed part of the kinetic energy and converted into the deformation of the substrates; while Ni substrates might be too stiff so that the molten droplets could not adhere well to the substrate surface, and it resulted in a lower deposition efficiency. It seems plausible since SS has the intermediate value of hardness among the three, which resulted in not only a higher deposition efficiency but also more effective deformation of the molten droplets. On the other hand, the undulations could also be caused by the thermal expansion mismatch between the ceramic coatings and the substrates underneath upon cooling. This is because the rapid cooling of the molten droplets would induce a significant volume shrinkage of CeO_2_ coatings. This therefore would cause the severe deformation of Al substrates due to the highest coefficient of thermal expansion (CTE) among the three substrates as shown earlier in Table 2. More evidence could be revealed from the detailed observations of the coating cross-sectional microstructure.


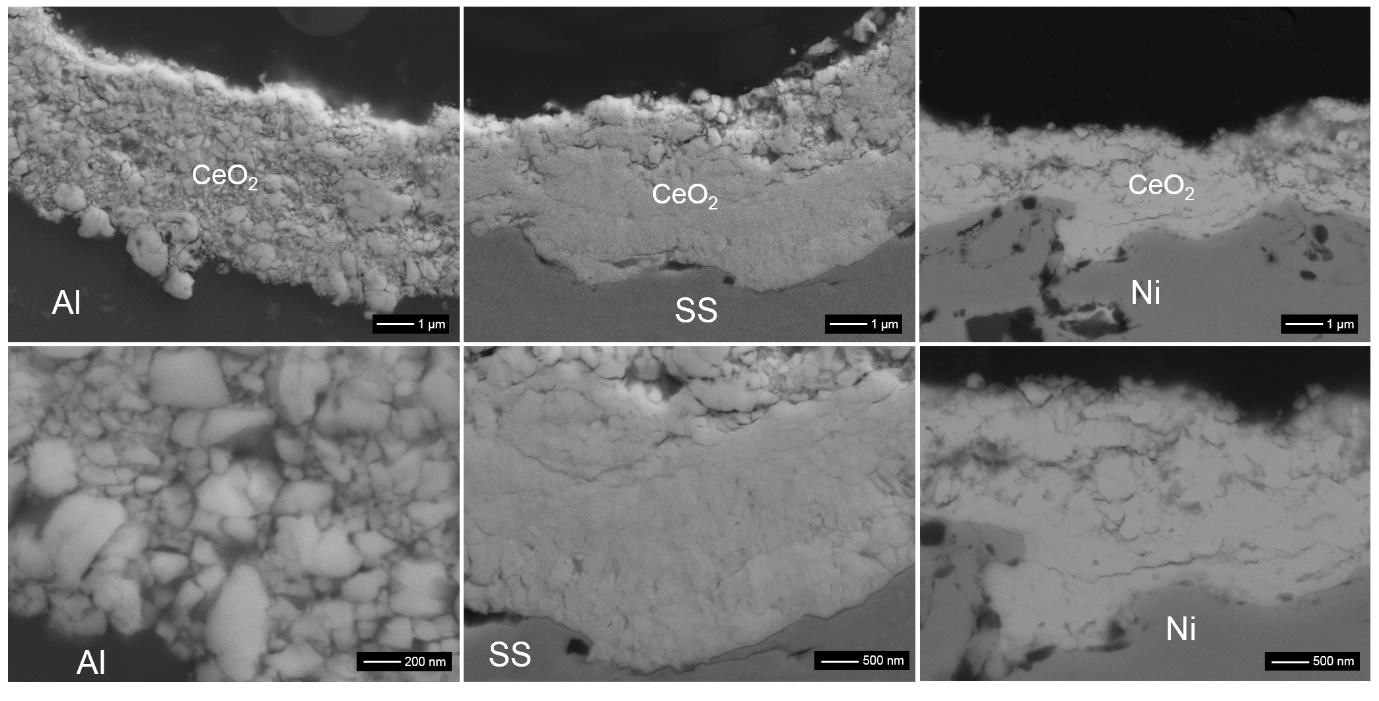


Figure S2. FEG-SEM images showing the cross-sectional microstructure of the as-sprayed CeO_2_ on Al, SS and Ni alloys under low (top row) and high (bottom row) magnifications.

Figure S2 shows the representative cross-sectional microstructure of the as-sprayed CeO_2_ coatings, which could further reveal the deposition mechanism. First of all, CeO_2_ (Al) coating shows distinctive microstructure which is composed of non-deformed particles, that are similar to the original feedstock powders as shown earlier in Figure 1, throughout the coatings. Meanwhile, CeO_2_ (SS) and CeO_2_ (Ni) coatings are observed to be much denser, especially near the coating/substrate interface, with negligible porosity indicating fully deformed and well sintered particles. For ceramic coatings, a higher density is generally an indicator of better mechanical properties^5^. Therefore, SS is seen to be the most ideal substrate among the three, in terms of deposition efficiency, as well as the coating density and microstructure.

*Phase analysis and crystallite size*


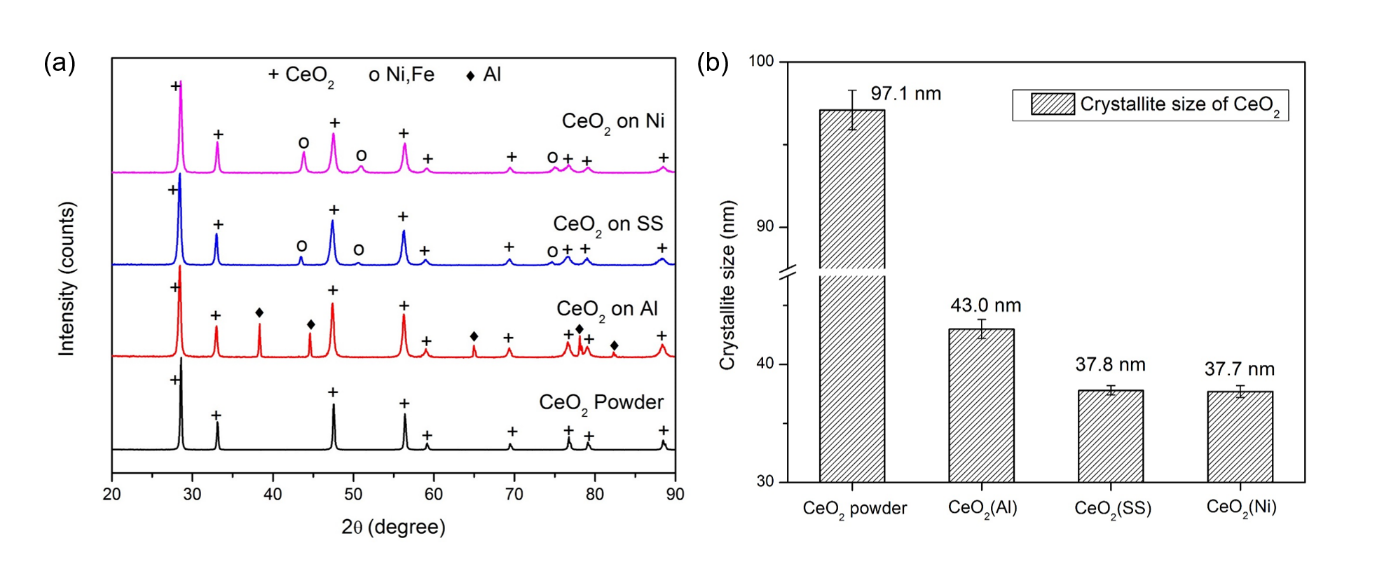


Figure S3. (a) XRD patterns of CeO_2_ powder and as-sprayed coatings on Al, SS and Ni substrates; and (b) the crystallite size of CeO_2_ phase as obtained from the Rietveld refinement.

Figure S3 (a) shows combinations of XRD patterns obtained from the feedstock powder and the as-sprayed coatings on the three substrates. First of all, it is observed that after spraying, all three coatings have fully retained the original CeO_2_ cubic phase from the feedstock powder with no phase transformation. Peaks from the substrates were also indexed since the coating thickness is less than the penetration depth of x-ray, which is generally several microns. Another prominent feature is the broadening of the CeO_2_ peaks of all the three coatings and it is believed to be caused by the reduction of crystallite size in suspension HVOF thermal sprayed coatings, which has an inversely correlation with the peak width^6,7^.

The crystallite size of the feedstock and as-sprayed coatings is plotted in Figure S3 (b), which was calculated by the coherent diffracting crystalline domains (CDD) as obtained by WPPM incorporated Rietveld Refinement of the whole XRD patterns for a higher accuracy rather than using empirical functions^6^. The crystallite size of feedstock powder is about 100 nm, which corresponds well with the particle size measurements showing a size range of 50 to 200 nm as shown earlier in Figure 1. A large particle could be composed of several nano-crystallites, which depends on the synthesis process of the feedstock submicron powder. In addition, a significant reduction of crystallite size is observed from feedstock powder to the as-sprayed coatings, which is a unique feature of suspension thermal sprayed technique attributing to the high particle velocity and rapid cooling so the crystallite growth could be effectively inhibited^3,4^. It is worth noting, however, the CeO_2_ (Al) has a slightly higher crystallite size (43.0 nm) than the other two with very close values (37.7~37.8 nm). It indicates a longer crystallite growth period, which could only occur during the solidification of the molten droplets after deposited on the Al substrates and also subjected to rapid cooling. Although it might not be possible to monitor the real-time temperature change on the substrates, it is highly possible that the crystallite growth of CeO_2_ (Al) was promoted by the extra heat generated by the melting of Al substrates due to a much lower melting point (640-650 °C) than the other two (1300-1400 °C, See Table 2). The melted Al substrates became even softer, which failed to provide sufficient mechanical resistance for the formation of fully-deformed particles and resulted in highly-deformed substrates and undesirable porous coating microstructure with non-deformed particles as shown earlier in Figure S2. These results have demonstrated that the current flame power is too high for the deposition of uniform and dense CeO_2_ coatings on the Al components or any other light alloys with low melting points and hardness.

Reference

1. Zhang, F., Robinson, B. W., de Villiers-Lovelock, H., Wood, R. J. & Wang, S. C. Wettability of hierarchically-textured ceramic coatings produced by suspension HVOF spraying. *Journal of Materials Chemistry A* **3**, 13864-13873 (2015).

2. Bolelli, G. *et al.* Microstructural and tribological investigation of high-velocity suspension flame sprayed (HVSFS) Al2O3 coatings. *Journal of thermal spray technology* **18**, 35-49 (2009).

3. Pawlowski, L. Suspension and solution thermal spray coatings. *Surface and Coatings Technology* **203**, 2807-2829 (2009).

4. Pawlowski, L. Finely grained nanometric and submicrometric coatings by thermal spraying: A review. *Surface and Coatings Technology* **202**, 4318-4328 (2008).

5. Bai, M., Maher, H., Pala, Z. & Hussain, T. Microstructure and phase stability of suspension high velocity oxy-fuel sprayed yttria stabilised zirconia coatings from aqueous and ethanol based suspensions. *Journal of the European Ceramic Society* **38**, 1878-1887 (2018).

6. Pala, Z., Shaw, E., Murray, J., Senin, N. & Hussain, T. Suspension high velocity oxy-fuel spraying of TiO 2: A quantitative approach to phase composition. *Journal of the European Ceramic Society* **37**, 801-810 (2017).

7. Bai, M., Khammas, R., Guan, L., Murray, J. W. & Hussain, T. Suspension high velocity oxy-fuel spraying of a rutile TiO2 feedstock: Microstructure, phase evolution and photocatalytic behaviour. *Ceram Int* **43**, 15288-15295 (2017).
